# Microleakage and bond strength analysis of zirconia-reinforced fiber posts using different resin cements in endodontically treated teeth

**DOI:** 10.6026/973206300212017

**Published:** 2025-07-31

**Authors:** Kanhu Keshav Mahapatra, Jasmine Marwaha, Philip Pradeep, Vipin Arora, Ashtha Arya, Shivani Agarwal

**Affiliations:** 1 Department of Conservative Dentistry and Endodontics, Kalinga institute of dental sciences, KIIT University, Patia, Bhubaneswar, Odisha, India; 2 Department of Conservative dentistry and Endodontics, National Dental College and Hospital, Derabassi-140507, Punjab, India; 3 Department of Restorative Dentistry, MAHSA University, SP2, BANDAR SAUJANA PUTRA, 42610, Selangor, Malaysia; 4 Department of Restorative Dental Sciences, Faculty of Dentistry, Taif University, Kingdom of Saudi Arabia; 5 Department of Conservative Dentistry and Endodontics, SGT Dental College, Hospital and Research Institute, SGT University, Gurugram, Haryana- 122505, India; 6 Department of Conservative Dentistry and Endodontic, Babu Banarasi Das College of Dental Sciences, Lucknow-226021, India

**Keywords:** zirconia-reinforced fiber posts, microleakage, push-out bond strength, resin cement, endodontically treated teeth

## Abstract

Microleakage and push-out bond strength of zirconia-reinforced fiber posts cemented with three resin cement systems in endodontically treated maxillary central incisors is of interest. Ninety extracted teeth were divided into three groups based on the use of self-adhesive, self-etch and conventional total-etch resin cements. Self-adhesive resin cements showed the least microleakage, indicating superior sealing ability. Total-etch systems exhibited the highest push-out bond strength, particularly in coronal regions. Bond strength decreased progressively from coronal to apical regions across all cement groups.

## Background:

Endodontically treated teeth require comprehensive restoration strategies due to substantial loss of coronal tooth structure and reduced fracture resistance compared to vital teeth [1, 2, 3-4]. The restoration of severely compromised endodontically treated teeth often necessitates the use of intracanal posts to provide adequate retention and support for core materials and final restorations [5]. Among various post systems available, fiber-reinforced composite posts have gained widespread acceptance due to their favorable mechanical properties, including an elastic modulus similar to dentin, which facilitates more uniform stress distribution along the root-structure [6]. The success of post-retained restorations depends critically on the establishment of an effective seal at the post-dentin interface to prevent microleakage, which represents the most significant cause of failure in endodontically treated teeth. Microleakage can lead to secondary caries, root canal contamination, and eventual restoration failure. Additionally, adequate bond strength between the post and root dentin is essential for long-term clinical success, as debonding represents a common mode of failure in fiber post systems [7].

Zirconia-reinforced fiber posts represent advancement in post technology, combining the favorable elastic properties of glass fiber posts with enhanced mechanical strength provided by zirconia particles. These posts exhibit superior fracture resistance while maintaining the aesthetic advantages of non-metallic post systems [8]. However, the bonding characteristics of zirconia-reinforced fiber posts to root dentin using different resin cement systems remain incompletely understood. Resin cements play a crucial role in establishing the bond between fiber posts and root dentin [9]. Three primary categories of resin cements are commonly employed: conventional total-etch systems requiring separate acid etching and bonding agent application, self-etch systems that combine acid conditioning and bonding in simplified procedures, and self-adhesive systems that bond directly to tooth structure without separate conditioning steps [10]. Each system presents distinct advantages and limitations regarding bond strength, microleakage resistance, and clinical application protocols. Previous investigations have demonstrated regional variations in bond strength along the root canal, with consistently higher values observed in coronal regions compared to middle and apical thirds [11]. Therefore, it is of interest to evaluate microleakage and bond strength analysis of zirconia-reinforced fiber posts using different resin cements in endodontically treated teeth.

## Materials and Methods:

## Specimen preparation:

Ninety freshly extracted human maxillary central incisors with fully formed apices and no visible cracks or defects were selected for this study following institutional review board approval. Teeth were stored in 0.5% chloramine solution at 4°C for no more than three months prior to use. All specimens were decoronated 2 mm coronal to the cemento-enamel junction using a high-speed diamond bur under water cooling. Root canal treatment was performed using standardized protocols. Access cavities were prepared, and working lengths were established 1 mm short of the apical foramen using size 10 K-files. Canal instrumentation was completed using rotary nickel-titanium files (ProTaper Next, Dentsply Sirona) to size X3 (30/.07 taper). Irrigation was performed with 2.5% sodium hypochlorite between each file, followed by final irrigation with 17% EDTA for 1 minute and 2.5% sodium hypochlorite. Canals were dried with paper points and obturated using gutta-percha and AH Plus sealer (Dentsply Sirona) via lateral condensation technique.

## Post space preparation:

After one week of storage at 37°C and 100% humidity, post spaces were prepared to 10 mm depth using manufacturer-provided drills, leaving 4-5 mm of apical gutta-percha. Post space preparation was completed under water cooling with intermittent drilling to prevent overheating. Final irrigation with 17% EDTA followed by saline was performed to remove debris.

## Experimental groups:

Specimens were randomly divided into three groups (n=30) based on the resin cement system used:

Group 1 (Self-Adhesive): TheraCem self-adhesive resin cement (Bisco Inc.) applied according to manufacturer's instructions without additional surface treatment [5].

Group 2 (Self-Etch): Panavia F2.0 with ED primer II (Kuraray Medical) following manufacturer's protocols for self-etch application [1].

Group 3 (Total-Etch): Variolink II with Excite DSC bonding agent (Ivoclar Vivadent) using conventional total-etch technique with 37% phosphoric acid etching for 15 seconds [1].

## Post cementation:

Zirconia-reinforced fiber posts (RelyX Fiber Post, 3M ESPE) were selected to fit post spaces with minimal binding. Post surfaces were cleaned with alcohol and silanized using Silane Primer (3M ESPE) for 1 minute prior to cementation. Cement was applied to both post surfaces and canal walls according to manufacturer's instructions. Posts were seated under finger pressure and excess cement was removed before light activation for 40 seconds from the coronal aspect.

## Microleakage assessment:

For microleakage evaluation, 15 specimens from each group were used. After cement polymerization, specimens were stored in distilled water at 37°C for 24 hours, then subjected to thermocycling (500 cycles, 5-55°C, 30-second dwell time). Root surfaces were sealed with nail varnish except for the coronal 2 mm, and specimens were immersed in 2% methylene blue solution for 24 hours. Specimens were sectioned longitudinally in buccolingual direction using a low-speed diamond saw under water cooling. Dye penetration was measured using a stereomicroscope at 20x magnification, with microleakage scored from 0-3: 0 = no penetration, 1 = penetration up to 1/3 of post length, 2 = penetration up to 2/3 of post length, 3 = penetration to apical extent of post.

## Push-out bond strength testing:

The remaining 15 specimens from each group were prepared for push-out testing. Each root was sectioned perpendicular to the long axis into 2 mm thick slices using a precision sectioning machine, yielding three sections representing coronal, middle, and apical regions [1, 10]. Section thickness was verified using digital calipers. Push-out testing was performed using a universal testing machine (Instron 5965) with a crosshead speed of 1 mm/minute. Cylindrical plungers of appropriate diameter (1.2 mm for coronal, 1.0 mm for middle, 0.8 mm for apical) were used to apply compressive force to the apical aspect of each section until post displacement occurred [10]. Bond strength values were calculated using the formula: Bond strength (MPa) = Load (N) / Bonded area (mm^2^), where bonded area = π x (r1 + r2) x h, with r1 and r2 representing coronal and apical post radii, and h representing section thickness.

## Statistical analysis:

Data were analyzed using SPSS version 28.0. Microleakage scores were analyzed using Kruskal-Wallis and Mann-Whitney U tests. Push-out bond strength data were evaluated using three-way ANOVA with cement type, root region, and their interaction as factors, followed by Tukey's post hoc tests. Statistical significance was set at p < 0.05.

## Results:

Microleakage assessment revealed significant differences among cement systems (p < 0.001). Table 1 (see PDF) presents the distribution of microleakage scores for each group. The self-adhesive cement group demonstrated significantly lower microleakage scores compared to both self-etch (p = 0.023) and total-etch (p = 0.001) groups [4]. No significant difference was observed between self-etch and total-etch groups (p = 0.087). Three-way ANOVA revealed significant main effects for cement type (p < 0.001), root region (p < 0.001), and their interaction (p = 0.012). Table 2 (see PDF) summarizes the push-out bond strength values. The total-etch system demonstrated significantly higher bond strength than both self-etch (p = 0.008) and self-adhesive (p < 0.001) systems. Self-etch cement showed significantly higher bond strength than self-adhesive cement (p = 0.003) [1]. Regional analysis revealed a consistent pattern across all cement systems, with coronal regions exhibiting the highest bond strength, followed by middle and apical regions. The decrease from coronal to apical regions was most pronounced in the total-etch group (36% reduction) compared to self-etch (32% reduction) and self-adhesive (35% reduction) groups [3]. Examination of post-push-out specimens revealed predominantly adhesive failures at the cement-dentin interface (78% of specimens), with mixed adhesive-cohesive failures accounting for 18% and pure cohesive failures within cement representing 4% of cases. No significant differences in failure mode distribution were observed among cement systems (p = 0.156) (Table 3 - see PDF).

## Discussion:

The results of this investigation demonstrate significant differences in both microleakage and bond strength characteristics among the three resin cement systems evaluated, leading to rejection of the null hypothesis. These findings have important implications for clinical decision-making in post-retained restorations of endodontically treated teeth. The superior microleakage resistance demonstrated by the self-adhesive cement system aligns with previous research highlighting the sealing capabilities of these materials [4, 5]. Self-adhesive cements achieve bonding through chemical interaction with calcium in hydroxyapatite and mechanical interlocking with surface irregularities, without requiring separate conditioning steps. The simplified application protocol reduces technique sensitivity and potential for moisture contamination, factors that can compromise seal integrity in conventional systems. The TheraCem system employed in this study incorporates MDP (10-methacryloyloxydecyl dihydrogen phosphate) monomer, which forms stable chemical bonds with calcium in tooth structure [5]. Additionally, the material's transition from acidic to alkaline pH during setting may contribute to improved sealing by promoting mineral precipitation at the interface [13, 14]. The intermediate microleakage performance of the self-etch system reflects the balance between simplified application and chemical bonding potential. Panavia F2.0 utilizes MDP monomer for chemical adhesion to dentin while maintaining some demineralization capability for micromechanical retention [1]. However, the self-etch approach may result in incomplete removal of the smear layer in deeper regions of the canal, potentially compromising penetration and sealing. The highest microleakage scores observed with the total-etch system, despite its superior bond strength, underscore the complex relationship between these parameters. While phosphoric acid etching creates deeper demineralization and potentially stronger micromechanical bonding, it also increases technique sensitivity and susceptibility to moisture contamination during the multi-step application process [13, 15 and 16]. The superior push-out bond strength demonstrated by the total-etch system confirms the effectiveness of aggressive surface conditioning in creating strong micromechanical bonds. Phosphoric acid etching removes the smear layer completely and creates deep demineralization of dentin, facilitating intimate penetration of bonding agents and resin cements [1]. The Variolink II/Excite DSC system employed utilizes HEMA and Bis-GMA monomers that can effectively penetrate the demineralized dentin matrix and form strong resin tags within dentinal tubules. The intermediate bond strength values observed with the self-etch system reflect the more conservative conditioning approach that partially dissolves the smear layer while preserving underlying dentin structure. This approach may result in a more stable bonding interface over time, as it avoids the potential for collagen degradation associated with aggressive acid etching [16, 17-18].

The lower bond strength values of the self-adhesive system, while concerning from a mechanical perspective, must be interpreted in context of the improved sealing properties and simplified application. These cements rely primarily on chemical bonding and limited micromechanical retention, which may be sufficient for clinical success when combined with excellent sealing capabilities [19, 20]. The consistent pattern of decreasing bond strength from coronal to apical regions across all cement systems confirms previous observations regarding anatomical and morphological factors affecting bonding in root canals [1, 3]. Several factors contribute to this pattern. Dentinal tubule density decreases significantly from coronal to apical regions, reducing the surface area available for micromechanical bonding. Additionally, the diameter of dentinal tubules decreases apically, limiting penetration of bonding agents and cements. C-factor (configuration factor) considerations become increasingly unfavorable in the apical region due to the high ratio of bonded to unbonded surfaces, resulting in increased polymerization stress that can compromise bond integrity [3, 21 and 22]. Accessibility for adequate moisture control and light penetration decreases in apical regions, potentially compromising polymerization and bonding effectiveness of light-activated components. The presence of residual moisture and anatomical complexity in apical regions may interfere with optimal cement adaptation and polymerization [23]. The findings of this study suggest that cement selection should consider both sealing requirements and mechanical demands. For situations where sealing is paramount, such as in teeth with questionable apical seals or high caries risk, self-adhesive cements may provide optimal performance. Conversely, when maximum retention is required, such as in short clinical crowns or high-stress situations, total-etch systems may be preferred despite their increased technique sensitivity. The superior performance of self-etch systems in balancing both microleakage resistance and bond strength makes them attractive for routine clinical use, particularly when combined with their simplified application protocols [1]. This *in vitro* study employed standardized laboratory conditions that may not fully replicate the clinical environment. Factors such as pulp chamber moisture, blood contamination, and dynamic loading conditions in the oral cavity could influence the relative performance of these systems. Future investigations should evaluate the long-term performance of these cement systems under cyclic loading conditions and assess the impact of aging on both microleakage and bond strength characteristics. Additionally, clinical studies are needed to validate these laboratory findings and assess the correlation between *in vitro* performance and clinical success rates. The use of extracted teeth with standardized root canal anatomy may not reflect the anatomical variations encountered clinically, including calcified canals, irregular morphology, and previous endodontic treatments.

## Conclusion:

Self-adhesive resin cements showed superior sealing ability, while total-etch systems provided the highest bond strength. Bond strength decreased from coronal to apical regions across all groups. Cement selection should be guided by clinical priorities-sealing, retention, or ease of use.
